# Supplementary material for: DNA-sensing inflammasomes cause recurrent atherosclerotic stroke
Source: Nature. 2024 Aug 7;633(8029):433–41. doi: 10.1038/s41586-024-07803-4 (PMC11390481; doi:10.1038/s41586-024-07803-4)
Supplement: Supplementary file 1 — Raw membrane and gel photographs for all protein analysis in the study. [file 41586_2024_7803_MOESM1_ESM.docx]

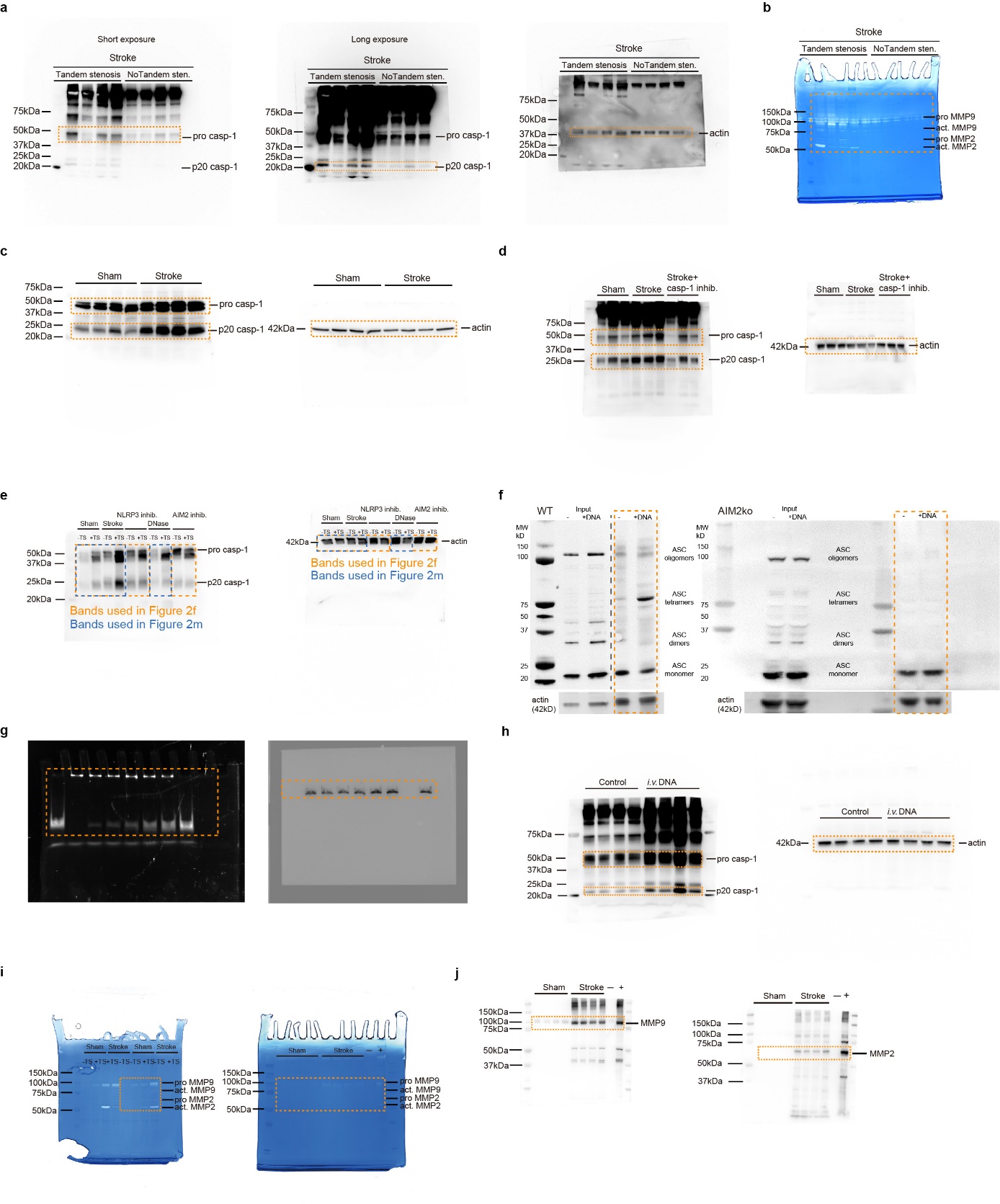

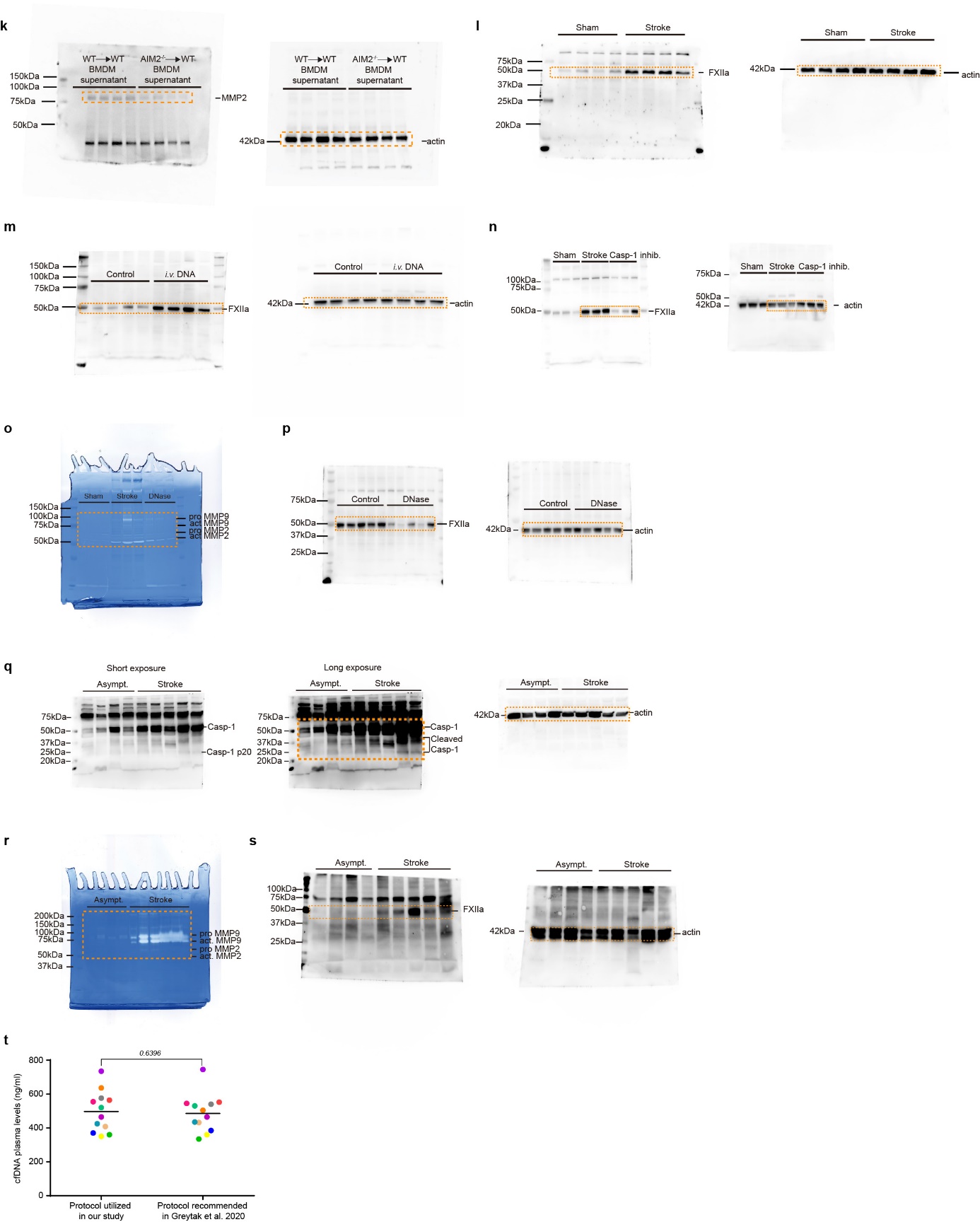


**Supplementary information 1. Raw membrane and gel photographs. a.** Caspase-1 cleavage SDS PAGE (4-12% gradient running gel) of CCA lysates 7d after stroke with or without CCA tandem stenosis ligation. ‘Short exposure’ was used for pro-Casp1 and ‘Long exposure’ for Casp1 p20. Cropped membranes were used in Extended Data Fig. 2j. **b**. MMP2 and MMP9 zymography microphotograph of CCA lysates 7d after stroke with or without tandem stenosis ligation. Cropped microphotograph was used in Extended data Fig. 2j. **c**. Caspase-1 cleavage SDS PAGE (12.5% running gel) of CCA lysates 7d after stroke or sham surgery. Cropped membranes were used in Extended Data Fig. 5a. **d**. Caspase-1 cleavage SDS PAGE (4-12% gradient running gel) of CCA lysates 7d after sham or stroke (± VX765 treatment) surgery. Cropped membranes were used in Extended Data Fig. 5e. **e.** Caspase-1 cleavage SDS PAGE (12.5% running gel) of CCA lysates 7d after sham or stroke (± MCC950; ± rhDNase I; ± Calixarene) surgery. Cropped membrane without ± rhDNase I was used in Fig. 2f; Same membrane with different cropping was used in Fig. 2m, with the first 4 lanes as controls used for both figure panels. **f**. ASC oligomerization SDS PAGE (15% running gel) of WT and *AIM2^−/−^* BMDMs stimulated with or without cell-free DNA (cfDNA). Cropped membranes were used in Fig. 2k. **g**. Raw EMSA gel (DNA detection) and Western blot gel (AIM2 detection) photographs for different Calixarene concentrations (0-1000 µM) interfering with the AIM2-dsDNA complex resulting in increased free DNA. **h.** Caspase-1 cleavage SDS PAGE (4-12% gradient running gel) of CCA lysates 24h after i.v. cell-free DNA injection (5µg). Cropped membranes were used in Fig. 2i. **i.** Left: MMP2 and MMP9 zymography microphotograph of CCA lysates 7d after stroke or sham surgery. Cropped microphotograph was used in Extended Data Fig. 8a. Right: MMP2 and MMP9 zymography microphotograph of supernatant of BMDMs stimulated with sham or stroke serum. Cropped microphotograph was used in Fig. 4c. **j**. MMP9 (left) and MMP2 (right) SDS PAGE (4-12% gradient running gel) of supernatant of BMDMs stimulated with sham or stroke serum. Cropped membranes were used in Fig. 4c. **k**. MMP2 SDS PAGE (4-12% gradient running gel) of BMDM lysates stimulated with supernatant from NET DNA-stimulated WT or AIM2-deficient BMDMs. Cropped membranes were used in Extended Data Fig. 8j. **l.** Activated factor XII (F. XIIa) SDS PAGE (12.5% running gel) of CCA lysates 7d after stroke or sham surgery. Cropped membranes were used in Extended Data Fig. 9a. **m**. F. XIIa SDS PAGE (4-12% gradient running gel) of CCA lysates 24h after an i.v. DNA or control bolus. Cropped membranes were used in Extended Data Fig. 9b. **n**. F. XIIa SDS PAGE (4-12% gradient running gel) of CCA lysates 7d after sham or stroke (± VX765 treatment) surgery. Cropped membranes were used in Extended Data Fig. 9c. **o.** MMP2 and MMP9 zymography microphotograph of CCA lysates 24h after sham or stroke (± rhDNase I treatment) surgery. Cropped microphotograph was used in Extended Data Fig. 9d. **p**. F. XIIa SDS PAGE (4-12% gradient running gel) of CCA lysates 24h after sham or stroke (± rhDNase I treatment) surgery. Cropped membranes were used in Extended Data Fig. 9d. **q.** Human caspase-1 cleavage SDS PAGE (12.5% running gel) of CCA lysates from asymptomatic or stroke patients. ‘Short exposure’ was used for pro-Casp1 and ‘Long exposure’ for Casp1 p20. Cropped membranes were used in Fig. 5d. **r.** Human MMP2 and MMP9 zymography microphotograph of CCA lysates from asymptomatic or stroke patients. Cropped microphotograph was used in Fig. 5h. **s.** Human F. XIIa zymography microphotograph (12.5% running gel) of CCA lysates from asymptomatic or stroke patients. Cropped microphotograph was used in Extended Data Fig. 10d. Cropped membranes used as panels in main and supplementary figures are indicated in orange boxes. Actin controls were all performed on the same membrane as the initial detection. **t.** Comparison of cfDNA isolation protocols. Murine plasma, from full blood withdrawn 4h after experimental stroke, was processed following our study protocol (see methods section) compared to the protocol recommended by Greytak et al. 2020 (n=12 per group; Wilcoxon signed rank test).
