# Supplementary material for: DNA-sensing inflammasomes cause recurrent atherosclerotic stroke
Source: Nature. 2024 Aug 7;633(8029):433–41. doi: 10.1038/s41586-024-07803-4 (PMC11390481; doi:10.1038/s41586-024-07803-4)
Supplement: Supplementary file 2 — Reporting Summary [file 41586_2024_7803_MOESM2_ESM.pdf]

nature portfolio

Corresponding author(s): Arthur Liesz and Stefan Roth

Last updated by author(s): Apr 23, 2024

Reporting Summary

Nature Portfolio wishes to improve the reproducibility of the work that we publish. This form provides structure for consistency and transparency in reporting. For further information on Nature Portfolio policies, see our Editorial Policies and the Editorial Policy Checklist.

Statistics

For all statistical analyses, confirm that the following items are present in the figure legend, table legend, main text, or Methods section.

n/a	Confirmed	
☐	☒	The exact sample size (*n*) for each experimental group/condition, given as a discrete number and unit of measurement
☐	☒	A statement on whether measurements were taken from distinct samples or whether the same sample was measured repeatedly
☐	☒	The statistical test(s) used AND whether they are one- or two-sided<br>*Only common tests should be described solely by name; describe more complex techniques in the Methods section.*
☒	☐	A description of all covariates tested
☐	☒	A description of any assumptions or corrections, such as tests of normality and adjustment for multiple comparisons
☐	☒	A full description of the statistical parameters including central tendency (e.g. means) or other basic estimates (e.g. regression coefficient) AND variation (e.g. standard deviation) or associated estimates of uncertainty (e.g. confidence intervals)
☐	☒	For null hypothesis testing, the test statistic (e.g. *F*, *t*, *r*) with confidence intervals, effect sizes, degrees of freedom and *P* value noted<br>*Give P values as exact values whenever suitable.*
☒	☐	For Bayesian analysis, information on the choice of priors and Markov chain Monte Carlo settings
☒	☐	For hierarchical and complex designs, identification of the appropriate level for tests and full reporting of outcomes
☒	☐	Estimates of effect sizes (e.g. Cohen's *d*, Pearson's *r*), indicating how they were calculated

Our web collection on statistics for biologists contains articles on many of the points above.

Software and code

Policy information about availability of computer code

Data collection

Agilent 2100 Bioanalyzer were used to detect cfDNA fragment lengths, Carl Zeiss LSM880 confocal microscope, Carl Zeiss LSM980 confocal microscope, Carl Zeiss Axio Imager M2 epifluorescent microscope, Carl Zeiss Axiovert 200M, C epifluorescent microscope, Carl Zeiss Axio Observer Z1 microscope were used to collect microscopy data. Northern Light (Cytek Biosciences, USA) flow cytometer was used to collect flow cytometry data. IL-1 beta ELISA (MLB00C, R&D system) kit and iMark Microplate reader (BIO-RAD, Germany) were used for IL-1 beta ELISA. Vevo3100LT (VisualSonics, Fujifilm) was used for ultrasonographic analyses. 3T nanoScan PET/MR (Mediso, Budapest) small animal scanner was used for MRI imaging. Fluoro-Jade C Ready-to-Dilute Staining Kit (TR-100-FJ, Biosensis) was used to detect degenerative neurons. Click-iT Plus TUNEL Assay for In Situ Apoptosis Detection, Alexa Fluor 647 dye kit (C10619, Thermofisher) was used to detect apoptotic cells. Picro-Sirius Red Stain Kit (ab245887, Abcam) was used for collagen analysis. Pierce BCA protein assay kit (23227, Thermofisher), Mini-PROTEAN Tetra Vertical Electrophoresis cell (BIO-RAD, Germany) and Mini Trans-Blot cell (BIO-RAD, Germany) were used for immunoblot analysis. Fusion Fx7 imaging system (Vilber, Germany) was used to acquire immunoblot images. Neutrophil isolation kit (130-097-658, Miltenyi Biotec) was used to isolate neutrophils. Plasma/serum Cell-free circulating DNA purification kit (#55100, NORGEN Biotek, Canada), Nanodrop Spectrophotometer (1000ND, Peqlab, USA), HS dsDNA Assay kit (Q32851, Thermofisher) were used to enrich and measure the cell-free DNA in bloodstream. Novex TM 10% Zymogram Plus (Gelatin) Protein Gels (ZY00100BOX, Thermofisher), colloidal blue staining kit (LC6025, Invitrogen) were used for MMP2, MMP9 gelatin zymography analysis.

Data analysis

Fiji, Image J v2 - Open source; VevoLab v3.2.0 software was used for ultrasound imaging analysis; ZEN 2 (blue edition) was used for image analysis; Cytek Northern Lights spectraflo software v3.0 (Cytek Biosciences, USA), FlowJo v. 10.6 (Treestar Inc.) software were used to analyse flow cytometry data. Nucline nanoScan 3.04.014.0000 softeware was used to process MRI images. Statistical analysis were performed using the GraphPad Prism (GraphPad Inc.). Rstudio Vers 1.1.477 was used for principal component analysis with ggplot2 package (Vers. 3.4.3).

For manuscripts utilizing custom algorithms or software that are central to the research but not yet described in published literature, software must be made available to editors and reviewers. We strongly encourage code deposition in a community repository (e.g. GitHub). See the Nature Portfolio guidelines for submitting code & software for further information.

Data

Policy information about availability of data

All manuscripts must include a data availability statement. This statement should provide the following information, where applicable:

- Accession codes, unique identifiers, or web links for publicly available datasets
- A description of any restrictions on data availability
- For clinical datasets or third party data, please ensure that the statement adheres to our policy

Any requests for raw data or reagents should be directed to and will be fulfilled by the corresponding authors, Arthur Liesz (Arthur.Liesz@med.uni-muenchen.de) and Stefan Roth (Stefan.Roth@med.uni-muenchen.de).

Research involving human participants, their data, or biological material

Policy information about studies with human participants or human data. See also policy information about sex, gender (identity/presentation), and sexual orientation and race, ethnicity and racism.

Reporting on sex and gender	Sex distribution was equally assigned in this study. Detailed information please see 'Patient corhots for epidemiological analysis', 'Patient cohort for carotid endarterectomy sample analysis' and 'Patient cohort for myocardial infarction sample analysis' in the Methods section.
Reporting on race, ethnicity, or other socially relevant groupings	Information on race/ethnicity was not recorded from study participants and was not used as analysis readout or proxy.
Population characteristics	Carotid endarterectomy samples of symptomatic or asymptomatic patients were collected at the Department and Cardiothoracic-Transplantation- and Vascular Surgery at Hannover Medical School between June 2018 and December 2020. . Carotid stenosis was defined as symptomatic if cerebral ischemia occurred in the territory of the affected artery and concurrent stroke etiologies were excluded following standardized stroke diagnostics including cranial computed tomography (CT) and/or magnetic resonance (MR) imaging. CT or MR-angiography, transthoracic or transesophageal echocardiography, cardiac rhythm monitoring and Doppler/duplex ultrasound. Peripheral venous blood was drawn immediately prior to surgery and EDTA whole blood samples were used for flow cytometry analysis. Carotid plaque samples were obtained during carotid endarterectomy and immediately preserved in phosphate-buffered saline. Both blood and tissue samples were sent for further analysis on the same day of collection. All patients provided written informed consent and the ethics committee at Hannover Medical School approved the study.Thirteen patients with symptomatic and seven patients with asymptomatic, high-grade carotid stenosis were recruited. Median age was 73 years (25th-75th percentile: 62-80 years). STEMI patients were prospectively recruited between September 2016 and February 2018 at the German Heart Centre Munich and the Klinikum rechts der Isar (both at the Technical University of Munich).<br>The diagnosis of STEMI was based on chest pain within the last 12 hours, persistent ST-segment elevation ≥ 1mm in at least 2 extremities or ≥ 2mm in at least 2 chest leads and diagnosis of type 1 myocardial infarction according to cardiac catheterization. Exclusion criteria were: cardiogenic shock, LV-EF ≤ 35, co-existing chronic or inflammatory diseases, anti-inflammatory drug therapy (e.g. cortisol), myocardial infarction type 2 – 5. Blood samples for plasma analysis were collected in EDTA tubes immediately after admission to the hospital or latest 6 hours after coronary intervention.<br>Age- and sex-matched patients with known stable coronary artery disease served as controls. They were prospectively recruited between February 2017 and February 2018 during consultation in the outpatient department of the German Heart Centre Munich for routine examination. Exclusion criteria were: history of myocardial infarction, reduced LV-EF, chronic or inflammatory diseases, anti-inflammatory drug therapy.<br>Blood samples for plasma analysis were collected in EDTA tubes on the day of consultation in the outpatient department. All patients provided written informed consent and the institutional ethics committee at Technical University Munich approved the study (235/16 S). EDTA tubes of both STEMI and control patients were centrifuged at 4°C and 2000xg for 15 minutes directly after collection. Plasma aliquots were stored at -80°C for further analysis.
Recruitment	Hannover: Patients were prospectively recruited at the Department of Neurology and Cardiothoracic-Transplantation- and Vascular Surgery at the Hannover Medical School between June 2018 and December 2020. All patients provided written informed consent to participate in the study.<br>Stroke blood samples/DEMDAS/PROCIS: Patients over 18y with an acute stroke (< 72h) were recruited through local certified stroke units. All patients provided written informed consent to participate in the study. Exclusion criteria were stroke onset >72h and hemorrhagic stroke.<br>STEMI samples: STEMI patients were prospectively recruited between September 2016 and February 2018 at the German Heart Centre Munich and the Klinikum rechts der Isar (both at the Technical University of Munich).<br>The diagnosis of STEMI was based on chest pain within the last 12 hours, persistent ST-segment elevation ≥ 1mm in at least 2 extremities or ≥ 2mm in at least 2 chest leads and diagnosis of type 1 myocardial infarction according to cardiac catheterization. Exclusion criteria were: cardiogenic shock, LV-EF ≤ 35, co-existing chronic or inflammatory diseases, anti-inflammatory drug therapy (e.g. cortisol), myocardial infarction type 2 – 5. Blood samples for plasma analysis were collected in EDTA tubes immediately after admission to the hospital or latest 6 hours after coronary intervention.
Ethics oversight	Stroke blood samples and PROCIS/DEDEMAS: The local ethics committee of the University Hospital of Munich (Number 17-005, 20-0935, 20-0935 and 121-09) approved the study and written informed consent for permission was given by all patients.<br>STEMI samples: All patients provided written informed consent and the institutional ethics committee at Technical University

Munich approved the study (235/16 S).
Endarterectomy samples: This study was approved by the ethics committee at Hannover Medical School (Ethics vote No. 7484-2017) and conducted in accordance with the ethical principles outlined in the Declaration of Helsinki.

Note that full information on the approval of the study protocol must also be provided in the manuscript.

Field-specific reporting

Please select the one below that is the best fit for your research. If you are not sure, read the appropriate sections before making your selection.

☒ Life sciences ☐ Behavioural & social sciences ☐ Ecological, evolutionary & environmental sciences

For a reference copy of the document with all sections, see nature.com/documents/nr-reporting-summary-flat.pdf

Life sciences study design

All studies must disclose on these points even when the disclosure is negative.

Sample size	1. In vivo: For this exploratory study, animal numbers were estimated based on previous results from the transient ischemia-reperfusion stroke model on extent and variability of atheroprogression after stroke.<br>2. In vitro: For the in vitro experiments, sample size was estimated based on previous in vitro studies. 3 biological independent experiments were considered with at least duplicates for technical replicates.<br>3. Hannover: Exploratory study with completely novel approach, no pre-emptive, dedicated sample size calculation was possible given the lack of prior data.<br>4. DEMDAS/PROCIS: Both studies are observational hospital-based cohort studies in patients suffering from acute stroke with no interventional group. No a priori calculation was made.<br>5. Myocard Infarct: This samples were used in exploratory study to unravel differences in cfDNA after myocardial infarction. No historical data was available to perform an a priori calculation.<br>6. DNA methylation: This experiment was as well planned as an exploratory study since no methylation analysis for post-stroke cfDNA was made before.
Data exclusions	For ischemia-reperfusion stroke model, data were excluded: 1. Insufficient MCA occlusion (a reduction in blood flow > 20% of the baseline value). 2. Death during the surgery. 3. Lack of brain ischemia as quantified post-mortem by histological analysis. For carotid tandem stenosis model, mice were excluded: 1. Death during the surgery. 2. No sign of atherosclerotic plaque formation as analyzed by histological analysis.
Replication	All attempts for replication were successful. At least 4 biological replicates were used to confirm reprodicibility.
Randomization	1. In vivo: Animals were randomly assigned to different groups.<br>2. In vitro: Grouping for in vitro (murine neurophils and macrophages) was not randomized.<br>3. Hannover: The clinical part of the work is observational in nature, and therefore no randomisation was carried out. Groups were defined according to whether patients were symptomatic or asymptomatic. Both groups are patient collectives with severe atherosclerotic disease and a correspondingly high level of vascular risk factors. Since we were explicitly interested in the possible effect of stroke, which is fulfilled as per definition by this group allocation, an adjustment for confounders is not only not necessary, but would even introduce additional bias.<br>4. DEMDAS/PROCIS: Both studies are observational hospital-based cohort studies in patients suffering from acute stroke with no interventional group. No randomization was used.<br>5. Myocard Infarct: Prospectively collected samples from STEMI or control patients were not randomized.<br>6. DNA methylation: Prospectively collected samples from acute ischemic stroke or control patients were not randomized.
Blinding	All analyses were performed by investigators blinded to group allocation during data collection.

Reporting for specific materials, systems and methods

We require information from authors about some types of materials, experimental systems and methods used in many studies. Here, indicate whether each material, system or method listed is relevant to your study. If you are not sure if a list item applies to your research, read the appropriate section before selecting a response.

Materials & experimental systems

n/a	Involved in the study	
☐	☒	Antibodies
☒	☐	Eukaryotic cell lines
☒	☐	Palaeontology and archaeology
☐	☒	Animals and other organisms
☒	☐	Clinical data
☒	☐	Dual use research of concern
☒	☐	Plants

Methods

n/a	Involved in the study	
☒	☐	ChIP-seq
☐	☒	Flow cytometry
☐	☒	MRI-based neuroimaging

Antibodies

Antibodies used

Immunostaining:
anti-CD68 (rat, ab53444, abcam, 1:200), anti-alpha smooth muscle actin (mouse, ab7817, abcam, 1:200), anti-Iba-1 (rabbit, 019-19741, Wako, 1:200), anti-Ki 67 (rabbit, 9129S, Cell Signaling, 1:200), anti-mouse caspase-1 (p20; mouse, AG-20B-0042-C100, Adipogen, 1:1000), recombinant anti-MMP2 (rabbit, ab92536, abcam, 1:1000), anti-MMP9 (rabbit, ab38898, abcam, 1:1000), anti-mouse actin (rabbit, A2066-.2ml, Sigma, 1:1000), anti-human caspase-1 (p20; mouse, AG-20B-0042B-C100, Adipogen, 1:1000), anti-Factor XII (rabbit, PA5-116703, Invitrogen, 1:100), anti-CD31 (rat, BM4086, OriGene, 1:200), anti-Von Willebrand Factor (sheep, ab11713, abcam, 1:50), anti-CD41 (rat, ab33661, abcam, 1:200), anti-Fibrinogen (rabbit, ab34269, abcam, 1:100), goat anti-rabbit IgG (H+L) Crossed-Absorbed Secondary Antibody, Alexa Fluor 594 (A-11012, Invitrogen, 1:200), goat anti-Mouse IgG (H+L) Highly Crossed-Absorbed Secondary Antibody, Alexa Fluor 488 (A-32723, Invitrogen, 1:200), goat anti-rat IgG (H+L) Crossed-Absorbed Secondary Antibody, Alexa Fluor 647 (A-21247, Invitrogen, 1:200), donkey anti-sheep IgG (H+L) Crossed-Absorbed Secondary Antibody, Alexa Fluor 594 (A-11016, Invitrogen, 1:200), anti-mouse IgG (goat, HRP-conjugated, P0447, Dako, 1:5000), anti-rabbit IgG (goat, HRP-conjugated, PI-1000, Vector, 1:5000). anti-mouse ASC (rabbit, AL177, 1:100), anti-mouse beta tubulin (mouse, T4027; 1:100). anti-mouse collagen I (rabbit, EPR24331-53, 1:250).
Flow cytometry:
anti-mouse CD45-APC-Cy7 (clone: 30-F11, 103116, Biolegend, 3 µg per mouse), anti-mouse CD45-eFluor450 (clone: 30-F11, 48-0451-82, Invitrogen, 3µg per mouse; 1:200), anti-mouse CD11b-PerCP-Cy5.5 (clone: M/70, 45-0112-82, Invitrogen, 1:200), anti-mouse Ly6G-PE-Fluor610 (clone: 1A8-ly6g, 61-9668-82, Invitrogen, 1:200), anti-mouse Ly6C-BV570 (clone: HK1.4, 128030, Biolegend, 1:200), anti-mouse CD192-APC (clone: SA203G11, 150628, Biolegend, 1:200), anti-mouse MHC II-PE (clone: NIMR-4, 12-5322-81, Invitrogen, 1:200), anti-mouse F4/80-PE-Cyanine7 (clone: BM8, 25-4801-82, Invitrogen, 1:200), anti-human CD3-FITC (clone: HIT3a, 11-0039-42, Invitrogen, 1:200), anti-human CD8a-PE (clone: SK1, 12-0087-42, Invitrogen, 1:200), anti-human CD19-APC (clone: HIB19, 17-0199-42, Invitrogen, 1:200), anti-human CD45-eFluor 450 (clone: 2D1, 48-9459-42, Invitrogen, 1:200), anti-human CD14-PerCP-Cy5.5 (clone: 61D3, 45-0149-42, Invitrogen, 1:200), anti-human CD16-FITC (clone: CB16, 11-0168-42, Invitrogen, 1:200), anti-human CD11b-PE (clone: ICRF44, 12-0118-42, Invitrogen, 1:200)
Antibody-based depletion:
anti-mouse Ly6G InVivoMab (clone: 1A8, BE0075-1, BioXCell, 14 mg kg-1 body weight); anti-mouse IgG non-reactive isotype control (BE0083, BioXCell, 14 mg kg-1 body weight), anti-mouse IL-1beta InvivoMab (clone: B122, BE0246, BioXCell, 4 mg kg-1 body weight), Polyclonal armenian hamster IgG InVivoMab (BE0091, BioXCell, 4 mg kg-1 body weight)

Validation

Immunostaining:
CD68 (rat, ab53444, abcam): mouse, immunofluorescence, https://www.abcam.com/cd68-antibody-fa-11-ab53444.html;
alpha smooth muscle actin (mouse, ab7817, abcam): mouse/human, immunofluorescence, https://www.abcam.com/alpha-smooth-muscle-actin-antibody-1a4-ab7817.html;
Iba-1 (rabbit, 019-19741, Wako): mouse/human, immunofluorescence, https://www.fujifilmcdi.com/anti-iba1-polyclonal-antibody-019-19741?gclid=CjwKCAiA_vKeBhAdEiwAFb_nrUda60XIg-w6G9L4Fm-qgsxqOmIH59dikDjcZDr7TgocUXwRKrWSoBoCSA8QAvD_BwE;
Ki 67 (rabbit, 9129S, Cell Signaling): mouse/human, immunofluorescence, https://www.cellsignal.com/products/primary-antibodies/ki-67-d3b5-rabbit-mab/9129?site-search-type=Products&N=4294956287&Ntt=9129s&fromPage=plp&_requestid=144354;
CD31 (rat, BM4086, OriGene): mouse, immunofluorescence, https://www.origene.com/catalog/antibodies/primary-antibodies/bm4086/pecam1-rat-monoclonal-antibody-clone-id-er-mp12;
Von Willebrand Factor (sheep, ab11713, abcam): mouse/human, immunofluorescence, https://www.abcam.com/von-willebrand-factor-antibody-ab11713.html;
CD41 (rat, ab33661, abcam): mouse, immunofluorescence, https://www.abcam.com/cd41-antibody-mwreg30-ab33661.html;
Fibrinogen (rabbit, ab34269, abcam): mouse/human, immunofluorescence, https://www.abcam.com/fibrinogen-antibody-ab34269.html;
Factor XII (rabbit, PA5-116703, Invitrogen): mouse/human, immunoblot, immunofluorescence, https://www.thermofisher.com/antibody/product/Factor-XII-Antibody-Polyclonal/PA5-116703;
Mouse caspase-1 (p20; mouse, AG-20B-0042-C100, Adipogen): mouse, immunoblot, immunofluorescence, https://adipogen.com/ag-20b-0042-anti-caspase-1-p20-mouse-mab-casper-1.html/;
MMP2 (rabbit, ab92536, abcam): mouse/human, immunoblot, https://www.abcam.com/mmp2-antibody-epr1184-ab92536.html;
MMP9 (rabbit, ab38898, abcam): mouse, immunoblot, https://www.abcam.com/mmp9-antibody-ab38898.html;
Actin (rabbit, A2066-.2ml, Sigma): mouse/human, immunoblot, https://www.sigmaaldrich.com/DE/en/product/sigma/a2066;
Human caspase-1 (p20; mouse, AG-20B-0042B-C100, Adipogen): human, immunoblot, https://adipogen.com/ag-20b-0042b-anti-caspase-1-p20-mouse-mab-casper-1-biotin.html;
Goat anti-rabbit IgG (H+L) Crossed-Absorbed Secondary Antibody, Alexa Fluor 594 (A-11012, Invitrogen): mouse/human, immunofluorescence, https://www.thermofisher.com/antibody/product/Goat-anti-Rabbit-IgG-H-L-Cross-Adsorbed-Secondary-Antibody-Polyclonal/A-11012;
Goat anti-Mouse IgG (H+L) Highly Crossed-Absorbed Secondary Antibody, Alexa Fluor 488 (A-32723, Invitrogen): mouse/human, immunofluorescence, https://www.thermofisher.com/antibody/product/Goat-anti-Mouse-IgG-H-L-Highly-Cross-Adsorbed-Secondary-Antibody-Polyclonal/A32723;
Goat anti-rat IgG (H+L) Crossed-Absorbed Secondary Antibody, Alexa Fluor 647 (A-21247, Invitrogen): mouse/human, immunofluorescence, https://www.thermofisher.com/antibody/product/Goat-anti-Rat-IgG-H-L-Cross-Adsorbed-Secondary-Antibody-Polyclonal/A-21247;
Mouse IgG (goat, HRP-conjugated, P0447, Dako): mouse, immunoblot, https://www.agilent.com/en/product/specific-proteins/elisa-kits-accessories/goat-anti-mouse-immunoglobulins-hrp-affinity-isolated-2717109;
Rabbit IgG (goat, HRP-conjugated, PI-1000, Vector): rabbit, immunoblot, https://vectorlabs.com/products/antibodies/peroxidase-goat-anti-rabbit-igg.
Flow cytometry:
CD45 (APC-Cy7, clone: 30-F11, 103116, Biolegend): mouse, https://www.biolegend.com/en-us/products/apc-cyanine7-anti-mouse-cd45-antibody-2530?GroupID=BLG1932;
CD45 (eFluor450, clone: 30-F11, 48-0451-82, Invitrogen): mouse, https://www.thermofisher.com/antibody/product/CD45-Antibody-clone-30-F11-Monoclonal/48-0451-82;

CD11b (PerCP-Cy5.5, clone: M/70, 45-0112-82, Invitrogen): mouse, https://www.thermofisher.com/antibody/product/CD11b-Antibody-clone-M1-70-Monoclonal/45-0112-82;
Ly6G (PE-Fluor610, clone: 1A8-ly6g, 61-9668-82, Invitrogen): mouse, https://www.thermofisher.com/antibody/product/Ly-6G-Antibody-clone-1A8-Ly6g-Monoclonal/61-9668-82;
Ly6C (BV570, clone: HK1.4, 128030, Biolegend): mouse, https://www.biolegend.com/en-us/products/brilliant-violet-570-anti-mouse-ly-6c-antibody-7392;
CD192 (APC,clone: SA203G11, 150628, Biolegend): mouse, https://www.biolegend.com/en-us/products/apc-anti-mouse-cd192-ccr2-antibody-17676;
MHC II (PE, clone: NIMR-4, 12-5322-81, Invitrogen): mouse, https://www.thermofisher.com/antibody/product/MHC-Class-II-I-A-Antibody-clone-NIMR-4-Monoclonal/12-5322-81;
F4/80 (PE-Cyanine7, clone: BM8, 25-4801-82, Invitrogen): mouse, https://www.thermofisher.com/antibody/product/F4-80-Antibody-clone-BM8-Monoclonal/25-4801-82;
CD3 (FITC, clone: HIT3a, 11-0039-42, Invitrogen): human, https://www.thermofisher.com/antibody/product/CD3-Antibody-clone-HIT3a-Monoclonal/11-0039-42;
CD8a (PE, clone: SK1, 12-0087-42, Invitrogen): human, https://www.thermofisher.com/antibody/product/CD8a-Antibody-clone-SK1-Monoclonal/12-0087-42;
CD19 (APC, clone: HIB19, 17-0199-42, Invitrogen): human, https://www.thermofisher.com/antibody/product/CD19-Antibody-clone-HIB19-Monoclonal/17-0199-42;
CD45 (eFluor 450, clone: 2D1, 48-9459-42, Invitrogen): human, https://www.thermofisher.com/antibody/product/CD45-Antibody-clone-2D1-Monoclonal/48-9459-42;
CD14 (PerCP-Cy5.5, clone: 61D3, 45-0149-42, Invitrogen): human, https://www.thermofisher.com/antibody/product/CD14-Antibody-clone-61D3-Monoclonal/45-0149-42;
CD16 (FITC, clone: CB16, 11-0168-42, Invitrogen): human, https://www.thermofisher.com/antibody/product/CD16-Antibody-clone-eBioCB16-CB16-Monoclonal/11-0168-42;
CD11b (PE, clone: ICRF44, 12-0118-42, Invitrogen): mouse/human, https://www.thermofisher.com/antibody/product/CD11b-Antibody-clone-ICRF44-Monoclonal/12-0118-42.
ASC (clone: AL177, Rabbit, AG-25B-0006-C100, Adipogen): https://adipogen.com/ag-25b-0006-anti-asc-pab-al177.html/
Beta tubulin (clone: T4027; mouse, Sigma): https://www.sigmaaldrich.com/deepweb/assets/sigmaaldrich/product/documents/295/024/t4026dat.pdf
Collagen I (clone: EPR24331-53, rabbit, Abcam): https://www.abcam.com/products/primary-antibodies/collagen-i-antibody-epr24331-53-bsa-and-azide-free-ab279711.html
anti-mouse Ly6G InVivoMab (clone: 1A8, BioXCell); https://bioxcell.com/invivomab-anti-mouse-ly6g-be0075-1
anti-mouse IgG non-reactive isotype control (BioXCell), https://bioxcell.com/invivomab-mouse-igg1-isotype-control-unknown-specificity-be0083
anti-mouse IL-1beta InvivoMab (clone: B122, BioXCell), https://bioxcell.com/invivomab-anti-mouse-rat-il-1b
Polyclonal armenian hamster IgG InVivoMab (BioXCell), https://bioxcell.com/invivomab-polyclonal-armenian-hamster-igg-be0091

Animals and other research organisms

Policy information about studies involving animals; ARRIVE guidelines recommended for reporting animal research, and Sex and Gender in Research

Laboratory animals	All mice used in this study were between 6 to 20 weeks of age. C57BL/6J WT mice were purchased from Charles River and housed at the animal core facility of the Centre for Stroke and Dementia Research (Munich, Germany). Apoe-/- mice on C57BL/6J background were bred and housed at the animal core facility of the Centre for Stroke and Dementia Research (Munich, Germany). ApoE-/- (B6.129P2-Apoetm1Unc/J; JAX strain: 002052), wildtype (C57BL6/J; JAX strain: 000664), AIM2-/- (B6.129P2-Aim2Gt(CSG445)Byg/J; JAX strain: 013144), Pycard-/- (ASC-/-B6.129S5-Pycardtm1Vmd) and R26-CAG-ASC-citrine mice (B6.Cg-Gt(ROSA)26ortm1.1(CAG-Pycard/mCitrine*,-CD2*); JAX strain: 030744) were bred and housed at the animal core facility of the Centre for Stroke and Dementia Research (Munich, Germany). LDLr-/-Mx1Cre:c-Mybfl/fl mice were bred and housed at the animal facility of Walter Brendel Centre (Munich, Germany). ApoE-/-mice were fed an HFD (#88137, ssniff) from 8 weeks on. cGAS-/- (B6(C)-Cgastm1d(EUCOMM)Hmgu/J), NLRP1-/- (Del(11Nlrp1a-Nlrp1c-ps)1Smas) and NLRP3-/-C57BL6/J-NLRP3tm1Tsc) mice were bred and housed at the Gene Centre of the LMU University Munich (Germany).<br>All mice (besides ApoE-/-) were maintained on a standard rodent chow diet until used for experiments. Apoe-/- mice were maintained on a rodent chow diet for 8 weeks, and were fed a HFD (#88137, ssniff) containing 42% kcal fat from lard, 15% kcal protein, and 43% kcal carbohydrates from 8 weeks on. All mice were housed under specific pathogen free conditions in 12/12 h light/dark cycles, at 21 °C and 50% humidity with food and water.
Wild animals	This study did not involve wild animals.
Reporting on sex	Apoe-/- mice on C57BL/6J background were bred and housed at the animal core facility of the Centre for Stroke and Dementia Research (Munich, Germany). Both male and female mice were randomly assigned to different groups in this study.
Field-collected samples	This study did not involve samples collected from the field.
Ethics oversight	All animal experiments were performed in accordance with the guidelines for the use of experimental animals and were approved by the government committee of Upper Bavaria (Regierungspraesidium Oberbayern). Animal experiments were performed according to the guidelines of the Animal Research: Reporting of In Vivo Experiment (ARRIVE).

Note that full information on the approval of the study protocol must also be provided in the manuscript.

Plants

Seed stocks

Report on the source of all seed stocks or other plant material used. If applicable, state the seed stock centre and catalogue number. If plant specimens were collected from the field, describe the collection location, date and sampling procedures.

Novel plant genotypes

Describe the methods by which all novel plant genotypes were produced. This includes those generated by transgenic approaches, gene editing, chemical/radiation-based mutagenesis and hybridization. For transgenic lines, describe the transformation method, the number of independent lines analyzed and the generation upon which experiments were performed. For gene-edited lines, describe the editor used, the endogenous sequence targeted for editing, the targeting guide RNA sequence (if applicable) and how the editor was applied.

Authentication

Describe any authentication procedures for each seed stock used or novel genotype generated. Describe any experiments used to assess the effect of a mutation and, where applicable, how potential secondary effects (e.g. second site T-DNA insertions, mosiacism, off-target gene editing) were examined.

Flow Cytometry

Plots

Confirm that:

☒ The axis labels state the marker and fluorochrome used (e.g. CD4-FITC).

☒ The axis scales are clearly visible. Include numbers along axes only for bottom left plot of group (a 'group' is an analysis of identical markers).

☒ All plots are contour plots with outliers or pseudocolor plots.

☒ A numerical value for number of cells or percentage (with statistics) is provided.

Methodology

Sample preparation

Isolated CCA samples were mixed with digestion buffer, consisting of collagenase type XI (125 U/ml, C7657), hyaluronidase type 1-s (60 U/ml, H3506), DNase I (60 U/ml, D5319), collagenase type I (450 U/ml, C0130; all enzymes from Sigma Aldrich, Germany) in 1x PBS, and were digested at 750 rpm for 30 min at 37 °C. After digestion, CCA materials were homogenized through a 40 µm cell strainer, washed at 500 g for 7 min at 4 °C and resuspended in flow cytometry staining buffer (00-4222-26, ThermoFisher) to generate single cell suspensions. Cell suspension were incubated with according flow cytometry antibodies, and were washed and resuspended in FACS buffer before analysis. For each experiment, a compensation was developed using signle staining controls.
EDTA Blood was prepared using Histopaque Gradient (1500 xg for 30min). The buffy coat was isolated, washed at 500 g for 7 min at 4 °C and resuspended in flow cytometry staining buffer (00-4222-26, ThermoFisher) to generate single cell suspensions. Cell suspension were incubated with according flow cytometry antibodies, and were washed and resuspended in FACS buffer before analysis. For each experiment, a compensation was developed using signle staining controls.

Instrument

Data were acquired by a spectral flow cytometer (Nortnern Light, CYTEK, USA).

Software

Data were analyzed with FlowJo (v. 10.6).

Cell population abundance

Does not apply. No FACS sorting used.

Gating strategy

For all cell types, initial forward scatter versus side-scatter were adjusted to include all leukocyte. Forward scatter-A versus forward scatter-H were used to gate singlets. Dead cells were then excluded using Zombie NIR Fixable Viability Kit (423106, Biolegend). Cell populations were gated on live cells and defined as inflammatory monocytes: CD45+ CD11b+ Ly6Chigh CCR2 +; activated macrophages: CD45+ CD11b+ F4/80+ MCH II+; infiltrating leukocytes: CD45 eFluor450+ APC-Cy7-. Data were presented as percentage of specific cell populations or calculated as cell numbers from total live cells.

☒ Tick this box to confirm that a figure exemplifying the gating strategy is provided in the Supplementary Information.

Magnetic resonance imaging

Experimental design

Design type

Fixed interval imaging after stroke.

Design specifications

MRI was performed in a small animal scanner (3T nanoScan® PET/MR, Mediso, with 25 mm internal diameter quadrature mouse head coil) at 2 and 7 days after sham or stroke surgery. For scanning, mice were anesthetized with 1.2% isoflurane in 30 % oxygen/70 % air applied via face mask. Respiratory rate and body temperature (37 +/- 0,5 °C) were continuously monitored via an abdominal pressure sensitive pad and rectal probe and anaesthesia adjusted to keep them in a physiological range.
For Thrombus detection MRI was performed in a small animal scanner (BioSpec 7-T TEP-MRI system, and a surface coil (Bruker, Germany)) at 6 and 24h after NET DNA challenge. Mice were anesthetized using Isoflurane in a mixture of O2/

N2O (30/70) and kept under anesthesia during all the procedure, while maintaining a body temperature of 37°C. Prior to MRI, mice were subjected to caudal vein catheterization for DNA and M3P administration. Imaging data were obtained using a TOF sequence to visualize vascular structures, a T2*-weighted sequence for iron-sensitive imaging, and a T2-weighted sequence for tissue contrast. The MRI parameters were set at TR/TE = 12 ms/4.2 ms for the TOF sequence, TR/TE = 50 ms/8.6 ms for the T2*-weighted sequence, and TR/TE = 3500 ms/40 ms for the T2-weighted sequence. T2*-weighted images are presented as stack of 4 slices (minimum intensity), after bias fields correction using ImageJ software.

Behavioral performance measures

This study did not include behavioral performance measures.

Acquisition

Imaging type(s)

Structural, diffusion.

Field strength

Small animal scanner (3T nanoScan® PET/MR, Mediso, with 25 mm internal diameter quadrature mouse head coil). BioSpec 7-T TEP-MRI System, and a surface coil (Bruker, Germany)

Sequence & imaging parameters

The following sequences were obtained: coronal T2-weighted imaging (2D fast-spin echo (FSE), TR/TE = 3000/57.1 ms, averages 14, resolution 167 x 100 x 500 µm3), coronal T1-weighted imaging (2D fast-spin echo (FSE), TR/TE = 610/28,6 ms, averages 14, resolution 167 x 100 x 500 µm3)

For thrombus detection, MRI parameters were set at TR/TE = 12 ms/4.2 ms for the TOF sequence, TR/TE = 50 ms/8.6 ms for the T2*-weighted sequence, and TR/TE = 3500 ms/40 ms for the T2-weighted sequence

Area of acquisition

Mouse brain and carotid arteries

Diffusion MRI ☒ Used ☐ Not used

Parameters

DWI (2D spin echo (SE), TR/TE = 1439/50 ms , averages 4, resolution 167 x 100 x 700 µm3).

Preprocessing

Preprocessing software

Nucline nano Scan 3.04.014.0000; ImageJ software

Normalization

If data were normalized/standardized, describe the approach(es): specify linear or non-linear and define image types used for transformation OR indicate that data were not normalized and explain rationale for lack of normalization.

Normalization template

Describe the template used for normalization/transformation, specifying subject space or group standardized space (e.g. original Talairach, MNI305, ICBM152) OR indicate that the data were not normalized.

Noise and artifact removal

Describe your procedure(s) for artifact and structured noise removal, specifying motion parameters, tissue signals and physiological signals (heart rate, respiration).

Volume censoring

Define your software and/or method and criteria for volume censoring, and state the extent of such censoring.

Statistical modeling & inference

Model type and settings

Specify type (mass univariate, multivariate, RSA, predictive, etc.) and describe essential details of the model at the first and second levels (e.g. fixed, random or mixed effects; drift or auto-correlation).

Effect(s) tested

Define precise effect in terms of the task or stimulus conditions instead of psychological concepts and indicate whether ANOVA or factorial designs were used.

Specify type of analysis: ☒ Whole brain ☐ ROI-based ☐ Both

Statistic type for inference

(See Eklund et al. 2016)

Specify voxel-wise or cluster-wise and report all relevant parameters for cluster-wise methods.

Correction

Describe the type of correction and how it is obtained for multiple comparisons (e.g. FWE, FDR, permutation or Monte Carlo).

Models & analysis

n/a	Involved in the study
☒	☐ Functional and/or effective connectivity
☐	☒ Graph analysis
☒	☐ Multivariate modeling or predictive analysis

Graph analysis

Report the dependent variable and connectivity measure, specifying weighted graph or binarized graph, subject- or group-level, and the global and/or node summaries used (e.g. clustering coefficient, efficiency, etc.).
