# Supplementary material for: DNA-sensing inflammasomes cause recurrent atherosclerotic stroke
Source: Nature. 2024 Aug 7;633(8029):433–41. doi: 10.1038/s41586-024-07803-4 (PMC11390481; doi:10.1038/s41586-024-07803-4)
Supplement: Supplementary file 3 — Flow cytometry gating strategies for all analysis made in the study. [file 41586_2024_7803_MOESM3_ESM.pdf]

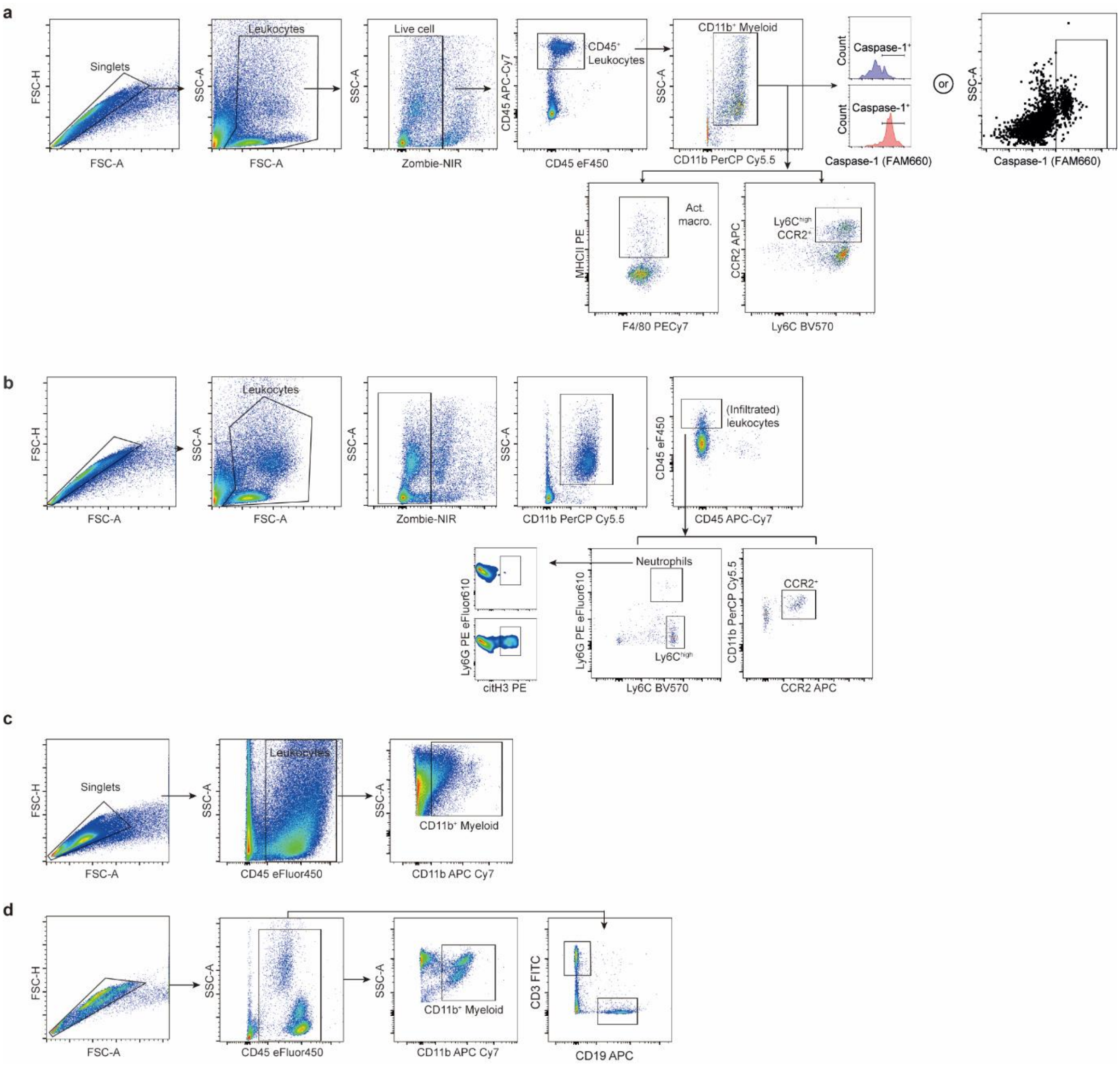

Supplementary information 2. Gating strategies for flow cytometry data. **a.** Gating strategy for murine flow cytometry analysis used in Fig. 1g, h, Fig. 2b, l, Fig. 3j, Extended Data Fig. 1d, Extended Data Fig. 4f, g, Extended Data Fig. 7g, h. **b.** Gating strategy for murine flow cytometry analysis used in Fig. 2d and Fig. 3b, i. **c, d.** Gating strategy for human flow cytometry analysis from (c) CCA plaque and (d) full blood used in Fig. 5b and Extended Data Figure 10 a-c.
