# Supplementary material for: DNA-sensing inflammasomes cause recurrent atherosclerotic stroke
Source: Nature. 2024 Aug 7;633(8029):433–41. doi: 10.1038/s41586-024-07803-4 (PMC11390481; doi:10.1038/s41586-024-07803-4)
Supplement: Supplementary file 4 — PROCIS and DEMDAS/DEDEMAS patient characteristics, found in Fig. 1 and Extended Data Fig. 1. [file 41586_2024_7803_MOESM4_ESM.pdf]

	PROSCIS	DEMDAS/DEDEMAS
Sample size (n)	1083	715*
Age (y), mean (SD)	67.6 (13.4)	68.0 (11.2)
Sex (% males)	59.7	66.7
TOAST subtype (%)		
Large artery atherosclerosis	20.9	26
Cardioembolism	24.1	23.4
Small artery occlusion	11.9	11.7
Other etiology	3.5	4.2
Unknown etiology	39.6	34.7

* 21 patients with hemorrhagic stroke originally included in the DEMDAS/DEDEMAS study have been excluded from the current analysis.

Supplementary table 1. Patient cohorts for epidemiological analysis (PROCIS and DEMDAS/DEDEMAS).
