# Supplementary material for: DNA-sensing inflammasomes cause recurrent atherosclerotic stroke
Source: Nature. 2024 Aug 7;633(8029):433–41. doi: 10.1038/s41586-024-07803-4 (PMC11390481; doi:10.1038/s41586-024-07803-4)
Supplement: Supplementary file 5 — Carotid Endarterectomy patient characteristics for (a)symptomatic carotid stenosis in Fig. 5. [file 41586_2024_7803_MOESM5_ESM.pdf]

	Symptomatic carotid stenosis (n=13)	Asymptomatic carotid stenosis (n=7)
Age (median (a); 25th-75th percentile)	64 (61-75)	78 (76-82)
Sex (male, n)	12 (92%)	6 (86%)
Arterial hypertension (n)	11 (84%)	7 (100%)
Diabetes mellitus (n)	4 (31%)	3 (43%)
BMI (median (kg/m²); 25th-75th percentile)	25.5 (23.2-29.3)	26.8 (24.5-31.6)
Current nicotine consumption (n)	5 (38%)	0 (0%)
Dyslipidemia (n)	9 (69%)	6 (85%)
Coronary artery disease	3 (23%)	3 (43%)
Peripheral artery disease	1 (8%)	2 (29%)
History of myocardial infarction	1 (8%)	1 (14%)
History of stroke	2 (15%)	0 (0%)

Supplementary table 2. Patient cohort characteristics for carotid endarterectomy samples.
