# Supplementary material for: DNA-sensing inflammasomes cause recurrent atherosclerotic stroke
Source: Nature. 2024 Aug 7;633(8029):433–41. doi: 10.1038/s41586-024-07803-4 (PMC11390481; doi:10.1038/s41586-024-07803-4)
Supplement: Supplementary file 6 — Myocardial Infarction cohort patient characteristics (MI versus Healthy control). [file 41586_2024_7803_MOESM6_ESM.pdf]

	Control (n=10)	STEMI (n=10)
Age (median (a); 25th-75th percentile)	65 (52-75)	52 (48-60)
Sex (male, n)	5 (50%)	7 (70%)
Arterial hypertension (n)	9 (90%)	7 (70%)
Diabetes mellitus (n)	4 (40%)	0 (0%)
BMI (median (kg/m²); 25th-75th percentile)	24.7 (23.5-28.7)	26.75 (23.2-28.1)
Current nicotine consumption (n)	2 (20%)	6 (60%)
Dyslipidemia (n)	9 (90%)	6 (60%)
Coronary artery disease	10 (100%)	10 (100%)
Peripheral artery disease	2 (20%)	4 (40%)
History of myocardial infarction	0 (0%)	1 (10%)
History of stroke	1 (10%)	0 (10%)

Supplementary table 3. Patient cohort characteristics for myocardial infarction.
