# Supplementary material for: DNA-sensing inflammasomes cause recurrent atherosclerotic stroke
Source: Nature. 2024 Aug 7;633(8029):433–41. doi: 10.1038/s41586-024-07803-4 (PMC11390481; doi:10.1038/s41586-024-07803-4)
Supplement: Supplementary file 7 — Comparison of cfDNA isolation protocols used in the study. [file 41586_2024_7803_MOESM7_ESM.pdf]

	**Protocol according to Greytak et al. 2020**	**Stroke (mouse)**	**MI (mouse)**	**ivDNA (mouse)**	**Stroke (human; CCA samples)**	**Stroke (human; cfDNA methylation)**	**MI (human)**
Time in EDTA (min)	≤120	5-15	15-30	5-15	≥180	15-30	≤15
1st centrifugation	800-1,600xg for 20min	3,000xg for 10min at 4°C	3,000xg for 10min at 4°C	3,000xg for 10min at 4°C	3,000xg for 15min	1,500xg for 10min at 4°C	1,600xg for 30 min
2nd centrifugation	14,000xg – 16,000xg for 10-20min at 4°C	3,000xg for 10min at 4°C	3,000xg for 10min at 4°C	3,000xg for 10min at 4°C	x	3000xg for 10min at 4°C	x
Storage	-80°C	-80°C	-80°C	-80°C	-80°C	-80°C	-80°C
Processing	Cat. 55100; NORGEN Biotek	Cat. 55100; NORGEN Biotek	Cat. 55100; NORGEN Biotek	Cat. 55100; NORGEN Biotek	Cat. 55100; NORGEN Biotek	QIAamp Blood DNA kit; symphony robot	Cat. 55100; NORGEN Biotek

Supplementary table 4. Comparison of cfDNA isolation protocols for human and murine plasma.
