# Supplementary material for: DNA-sensing inflammasomes cause recurrent atherosclerotic stroke
Source: Nature. 2024 Aug 7;633(8029):433–41. doi: 10.1038/s41586-024-07803-4 (PMC11390481; doi:10.1038/s41586-024-07803-4)
Supplement: Supplementary file 8 — Raw data cfDNA methylation pattern analysis in Figure 3a. [file 41586_2024_7803_MOESM8_ESM.pdf]

	GE/ml	GE/ml	GE/ml	GE/ml	GE/ml	GE/ml	GE/ml	GE/ml	GE/ml	GE/ml	GE/ml	
Samples	B cells	CD8	Monocytes	Neutrophils	NK	T cell	Tregs	Astrocyte	Neuron	Oligodendrocyte	Whole Brain	ng/ml
1903SS D1	7.2083965	0	83.2086796	189.259132	3.6906025	16.0505917	3.47467577	3.43660366	1.37973214	3.34689491	0	4.08
166g6M D1	24.8932491	9.07969485	214.928349	421.370643	24.6775148	112.003976	0.07076485	0	2.41098047	0	0	5.82
1775GI D1	25.1291815	10.4001178	137.958102	366.709418	31.9027386	142.411451	0	0	0	0.07756928	10.6528718	5.82
1606HA D3	27.3399231	6.90033212	232.433696	641.738379	29.4260358	44.6949264	0	4.92059673	1.59957859	0.01593612	NA	8.28
1669GM D3	58.1435244	9.61926388	535.390025	1403.37126	76.1606505	121.651995	22.6075025	21.2103412	3.1052947	0	23.4540614	28.56
94K_KE	13.3005175	45.4764273	165.28506	284.595363	32.8100854	125.776044	11.6587977	3.17723785	4.87215384	0	12.4023295	4.92
147WE D3	141.93347	66.6689757	1137.64417	4384.47637	31.068313	141.447863	57.3505471	55.6298196	8.19736115	10.5990742	0	54.4
634GG D1	20.3245305	8.31959674	94.1901312	321.910676	14.0745855	108.622441	2.82107711	4.36180027	0.06241501	0	0	6.52
144K_BH	8.40418308	0	9.61539192	442.355684	35.8747506	57.1471604	5.70994627	0	3.70789178	0	0	6.68
656GE D1	19.6150086	12.8916609	409.815036	548.169393	41.5433604	62.6328897	10.2496979	4.18977593	3.27376564	4.03809644	0	11.84
634GG D3	40.2235408	16.9715336	245.161395	1301.33228	5.49850058	65.5992407	4.62817559	23.6511252	1.21540804	6.77117446	0.75589852	14.16
172K_BK	33.1055337	10.720913	100.182236	1187.34025	10.3573401	127.789382	1.6634623	0.35017657	8.58073378	0	NA	12.96
656GE D3	40.3469764	34.9676045	1576.93991	2875.49844	58.3898883	133.087044	14.3289666	29.2397724	8.05092976	5.36384887	11.138101	45.2
877BA D1	16.1153889	14.8195991	179.595196	3328.57183	43.6535412	101.716713	15.7153527	1.39326855	0	0	0	19.4
851HH D1	1.65996635	8.27486972	114.014997	455.450034	29.8295399	52.8945139	0	3.84618806	0	0	0	4.56
888WH D1	10.5960957	8.70722777	232.080215	417.939079	23.486191	89.6117626	0	0.68726566	0	0	0	9.8
877BA D3	60.0739115	8.02293937	796.368077	6626.0416	95.9027061	93.4138477	10.0262213	3.70552569	0.75453078	0	0	43.2
9205M D1	5.59710864	3.32148322	218.540705	800.022326	83.4839001	88.5655757	0.10762043	0	0	0.11776805	0	9.32
641KE/2 D1	56.8288239	4.1804894	592.233019	1584.38274	27.6572556	141.270694	3.53804343	6.84642048	1.82190291	0	0	20.2
1600JS D1	143.718381	10.6604612	128.632126	333.120258	16.782232	60.7754899	0	0	0.01973596	0	1.10145928	5.58
1606HA D1	31.9321473	5.17880811	292.730635	792.427142	22.2875739	40.9344348	0	0.16921837	3.22809807	0	0	9.56
1481_DI D3	7.09503912	0.87373217	1.75463807	227.071892	31.5690205	15.0096806	6.02863593	NA	NA	NA	NA	4.32
1527TW D1	16.5990142	9.57597431	127.684298	421.463339	42.8820626	69.1774863	10.5466981	0	0	0	0	8.58
1463_BO D3	155.029114	92.9299321	2407.82406	9160.12962	111.743873	300.061271	85.5672792	99.7291263	16.2074581	24.2285006	25.1982989	72
1481D1 D1	32.7968116	13.1903815	128.236387	453.437031	8.56448438	78.272019	4.71051241	4.76294299	0	0.38416527	0	6.03
1425ME D3	59.857488	130.678292	1893.37817	4725.44313	289.344069	247.211681	77.868643	72.7451369	9.76368881	15.1040611	34.4849108	75.2
1463BO D1	40.6934479	22.6644914	554.760957	1805.90177	16.8991407	106.61555	8.90906986	12.5588589	2.39918834	0	3.54018481	17.07
1270KR D1	64.6314134	54.5361539	779.976134	780.037693	72.4317806	217.383419	5.36742857	9.99230219	0	0.84316422	0	17.36
	%	%	%	%	%	%	%	%	%	%	%	MEAN
Samples	B cells	CD8	Monocytes	Neutrophils	NK	T cell	Tregs	Astrocyte	Neuron	Oligodendrocyte	Whole Brain	

1903SS D1	0.58309038	0	6.73078687	15.3092548	0.29853447	1.29833946	0.28106806	0.27798839	0.11160714	0.27073181	0	25.1614014
166g6M D1	1.41161405	0.51487955	12.1878777	23.8945393	1.39938047	6.35137601	0.00401284	0	0.13671875	0	0	45.9003987
1775GI D1	1.424993	0.58975638	7.8231489	20.7948815	1.80909908	8.07568368	0	0	0	0.0043987	0.60408922	41.1260505
1606HA D3	1.08974359	0.27504074	9.26458827	25.5790875	1.17289408	1.78149768	0	0.19613035	0.0637577	0.0006352	NA	39.4233751
1669GM D3	0.67189363	0.11115807	6.18684796	16.2170459	0.88009553	1.40578337	0.26124727	0.24510198	0.03588409	0	0.27102991	26.2860877
94K_KE	0.8921971	3.05055323	11.0873018	19.0906224	2.20089655	8.43704175	0.78207074	0.21312873	0.32682349	0	0.83194676	46.9125825
147WE D3	0.86107959	0.40446622	6.90184049	26.5996674	0.18848472	0.85813351	0.34793333	0.33749405	0.04973161	0.06430229	0	36.6131332
634GG D1	1.02879844	0.421126	4.76776869	16.2946545	0.71243523	5.49831143	0.14279886	0.22078804	0.00315936	0	0	29.0898405
144K_BH	0.41521823	0	0.47505938	21.8550861	1.77243289	2.8234205	0.28210639	0	0.18319261	0	0	27.8065161
656GE D1	0.54675677	0.35934743	11.4233519	15.2798979	1.15799662	1.74585479	0.28570427	0.11678753	0.09125428	0.11255955	0	31.1195111
634GG D3	0.93750678	0.39556259	5.7140785	30.330692	0.12815584	1.52894876	0.10787081	0.5512466	0.02832802	0.15781858	0.01761804	39.8978265
172K_BK	0.84304928	0.27301351	2.55119168	30.2362244	0.26375494	3.25422172	0.04236091	0.00891742	0.21851276	0	NA	37.6912466
656GE D3	0.29459809	0.25531999	11.5142082	20.9957828	0.42634049	0.97175037	0.1046246	0.21349756	0.05878479	0.03916476	0.08132613	34.9553979
877BA D1	0.27415516	0.25211117	3.05527536	56.6256988	0.7426345	1.73040579	0.26734974	0.0237023	0	0	0	62.9713328
851HH D1	0.12014116	0.59889915	8.25191046	32.9634962	2.1589326	3.82827528	0	0.27837039	0	0	0	48.2000252
888WH D1	0.35684299	0.29323189	7.81572758	14.0748663	0.79094063	3.01784073	0	0.02314493	0	0	0	26.372595
877BA D3	0.45894383	0.06129247	6.08397565	50.6206576	0.73266338	0.71364937	0.07659685	NA	NA	NA	NA	58.7477792
9205M D1	0.19820071	0.11761793	7.73880313	28.3298037	2.95627063	3.1362192	0.00381098	0	0	0.00417032	0	42.4848966
641KE/2 D1	0.92848453	0.06830195	9.67606147	25.8860691	0.45187164	2.30811839	0.0578055	0.11185865	0.02976674	0	0	39.518338
1600JS D1	8.50032419	0.63052043	7.60803707	19.7026307	0.99259685	3.59460886	0	0	0.0011673	0	0.06514658	41.095032
1606HA D1	1.10237055	0.17878427	10.1057291	27.3563922	0.76941788	1.41315005	0	0.0058418	0.11144131	0	0	41.0431272
1481_DI D3	0.54203636	0.0667501	0.13404826	17.3475043	2.41176358	1.14668749	0.46056686	NA	NA	NA	NA	22.1093569
1527TW D1	0.63848747	0.36834354	4.91142566	16.2117496	1.64947505	2.66093865	0.4056828	NA	NA	NA	NA	26.8461028
1463_BO D3	0.71062117	0.42597145	11.036964	41.9881262	0.51221064	1.37541837	0.39222259	0.45713754	0.07429161	0.1110584	0.11550375	57.1995257
1481D1 D1	1.79502989	0.72193387	7.01861361	24.8174436	0.46875	4.28397173	0.25781502	0.26068464	0	0.02102607	0	39.6452685
1425ME D3	0.26269876	0.57351262	8.30953834	20.7387259	1.26985495	1.08494699	0.34174497	0.31925926	0.04285026	0.06628775	0.1513452	33.160765
1463BO D1	0.78677099	0.43819743	10.7258011	34.9154765	0.32672959	2.06131518	0.1722488	0.24281417	0.04638614	0	0.06844627	49.7841862
1270KR D1	1.22871541	1.03679324	14.8282181	14.8293884	1.37700911	4.13270175	0.10204082	0.18996483	0	0.01602949	0	37.7408611

Supplementary table 6. Raw data of cell-free DNA methylation analysis found in Figure 3a.
