# Supplementary material for: DNA-sensing inflammasomes cause recurrent atherosclerotic stroke
Source: Nature. 2024 Aug 7;633(8029):433–41. doi: 10.1038/s41586-024-07803-4 (PMC11390481; doi:10.1038/s41586-024-07803-4)
Supplement: Supplementary file 9 — Resource list of consumables and reagents for all methods. [file 41586_2024_7803_MOESM9_ESM.pdf]

REAGENT or RESOURCE	SOURCE	IDENTIFIER
**Antibodies**		
Anti-Ly6G *InVivo*MAb [1A8], Rat	BioXCell	BE0075-1
mouse IgG1 non-reactive isotype control, *InVivo*MAb, Mouse	BioXCell	BE0083
Anti-IL-1beta *InVivo*MAb [B122], Arm. Hamster	BioXCell	BE0246
polyclonal Armenian hamster IgG, *InVivo*MAb, Armenian hamster	BioXCell	BE0091
Anti-CD68 antibody [FA-11], Rat	Abcam	ab53444
Anti-alpha smooth muscle Actin antibody[1A4], Mouse	Abcam	ab7817
Anti Iba-1, Rabbit	Wako	019-19741
Anti-Ki-67 (D3B5) mAb, Rabbit	Cell Signaling	9129S
Anti-mouse caspase-1 (p20; CASPER1; mouse)	Adipogen	AG-20B-0042-C100
Recombinant Anti-MMP2 antibody	Abcam	ab92536
Anti-MMP9 antibody	Abcam	ab38898
Anti-ASC antibody, Rabbit	Adipogen	AL117
Anti-mouse actin, Rabbit	Sigma	A2066-.2ml
Anti-human caspase-1 (p20, BALLY-1; mouse)	Adipogen	AG-20B-004
Anti-Factor XII Polyclonal Antibody, Rabbit	Invitrogen	PA5-116703
Anti-CD31 Antibody, Rat	OriGene	BM4086
Anti-Von Willebrand Factor Antibody, Sheep	Abcam	ab11713
Anti-CD41 [MWReg30] Antibody, Rat	Abcam	ab33661
Anti-Fibrinogen Antibody, Rabbit	Abcam	ab34269
Anti-β-tubulin, Mouse	Sigma	T-4027
Anti-collagen I, Rabbit	Abcam	ab279711
Goat anti-Rabbit IgG (H+L) Crossed-Adsorbed Secondary Antibody, Alexa Fluor 594	Invitrogen	A-11005
Goat anti-Mouse IgG (H+L) Highly Cross-Adsorbed Secondary Antibody, Alexa Fluor 488	Invitrogen	A-32723

Goat anti-Rat IgG (H+L) Cross-Adsorbed Secondary Antibody, Alexa Fluor 647	Invitrogen	A-21247
Donkey anti-Sheep IgG (H+L) Cross-Adsorbed Secondary Antibody, Alexa Fluor 594	Invitrogen	A-11016
Anti-mouse IgG (goat, HRP-conjugated)	Dako	P0447
Anti-rabbit IgG (goat, HRP-conjugated)	Dako	PI-1000
Anti-mouse CD45 (APC-Cy7; 30-F11)	Biolegend	103116
Anti-mouse CD45 (eFluor450; 30-F11)	Invitrogen	48-0451-82
Anti-mouse CD11b (PerCP-Cy5.5; M/70)	Invitrogen	45-0112-82
Anti-mouse F4/80 (PE-Cyanie7; BM8)	Invitrogen	25-4801-82
Anti-mouse Ly6G (PE-Fluor610; 1A8-Ly6g)	Invitrogen	61-9668-82
Anti-mouse Ly6C (BV570; HK1.4)	Biolegend	128030
Anti-mouse CD192 (APC; SA203G11)	Biolegend	150628
Anti-mouse MHC II (PE; NIMR-4)	Invitrogen	12-5322-81
Anti-mouse Histone 3 (citrulline R2+R8+R17)	Abcam	ab5103
Anti-human CD3 (FITC; HIT3a)	Invitrogen	11-0039-42
Anti-human CD8a (PE; SK1)	Invitrogen	12-0087-42
Anti-human CD19 (APC; HIB19)	Invitrogen	17-0199-42
Anti-human CD45 (eFluor 450; 2D1)	Invitrogen	48-9459-42
Anti-human CD14 (PerCP-Cy5.5; 61D3)	Invitrogen	45-0149-42
Anti-human CD16 (FITC; CB16)	Invitrogen	11-0168-42
Anti-human CD11b (PE; ICRF44)	Invitrogen	12-0118-42
**Chemicals, peptides, and recombinant proteins**		
DMEM+GlutaMAX (4.5g/I D-Gluc. / Pyruvate)	Gibco	31966-021
Fetal calf serum (FCS)	Gibco	105000-064
Gentamycin (50mg/ml)	Gibco	15750-045
RPMI 1640 (L-Glutamine / 25 mM HEPES)	Gibco	52400-025
Penicillin / Streptomycin	Gibco	15140-122
LPS from E.coli	Adipogen	IAX-100-013-M001
Isopentane	Sigma	277258-1L

Phorbol 12-myristate 13-acetate (PMA)	Adipogen	AG-CN2-0010-M005
Recombinant Human DNase I	Roche	4716728001
Caspase-1 inhibitor (VX-765, Belnacasan)	Invivogen	Inh-vx765i-1
NLRP3 inflammasome inhibitor (MCC950)	Invivogen	Inh-mcc
Peptidylarginine Deiminase 4 (PAD4) inhibitor GSK484	Sigma	SML1658-5MG
**REAGENT**		
RIPA lysis buffer (+protease/phosphatase inhibitor)	Thermo Fisher Sci	8990
Cell lysis buffer 2	R&D system	895347
Zymogram Renaturing Buffer	Invitrogen	LC2670
Zymogram Developing Buffer	Invitrogen	LC2671
Collagenase type XI	Sigma	C7657
Hyaluronidase type 1-s	Sigma	H3506
DNase I	Sigma	D5319
Collagenase type I	Sigma	C0130
Ethylene diamine tetra acetic acid (EDTA)	Roth	Art.No.8043.2
Flow cytometry staining buffer	Thermo Fisher Sci	00-4222-26
Histopaque solution	Sigma	Histopaque-1119
DQ-gelatin	Invitrogen	D12054
Disuccinimidyl suberate	Thermo Fisher	21655
HEPES	Thermo Fisher	15630106
Magnesium chloride	Sigma	M8266
EGTA (egtazic acid)	Millipore	324626
Sucrose	Sigma	S0389
PMSF (phenylmethylsulfonyl fluoride)	Roche	10837091001
CHAPS	Thermo Fisher	28300
Kalium chloride	Sigma	P3911
4-sulfonic calixarene	Sigma	55523
**Critical commercial assays**		
Duoset ELISA murine IL-1beta	R&D system	MLB00C

Fluoro-Jade C Ready-to-Dilute Staining Kit	Biosensis	TR-100-FJ
Click-iT™ Plus TUNEL Assay for In Situ Apoptosis Detection, Alexa Fluor™ 647 dye	Thermo Fisher Sci	C10619
Picro-Sirius Red Stain Kit (Cardiac Muscle)	Abcam	Ab245887
Colloidal blue staining kit	Invitrogen	LC6025
Neutrophil isolation kit	Miltenyi Biotec	130-097-658
Plasma / Serum Cell-Free Circulating DNA Purification Mini Kit	NORGEN Biotek	55100
HS dsDNA Assay Kit	Thermo Fisher Sci	Q32851
FLICA 660 Caspase-1 Assay Kit	Immunochemistry Techn.	#9122
Agilent High Sensitivity DNA Kit	Agilent	5067-4626
DRAQ5 fluorescent probe	Thermo Fisher Sci	62251
SYTOX green	Thermo Fisher Sci	S7020
**Software**		
Cytek Northern lights	Cytek Biosciences	N/A
Microsoft Excel	Microsoft Corporation	N/A
FlowJo v. 10.6	Treestar Inc.	N/A
GraphPad Prism 6	Graphpad SoftwareInc	N/A

Supplementary table 6. Resource list for supplementary methods.
